# Antibacterial Activity Assessment of Chitosan/Alginate Lavender Essential Oil Membranes for Biomedical Applications

**DOI:** 10.3390/membranes14010012

**Published:** 2024-01-01

**Authors:** Encarnación Cruz Sánchez, María Teresa García, Ignacio Gracia, Soledad Illescas Fernández-Bermejo, Juan Francisco Rodríguez, Jesús Manuel García-Vargas, Dolors Vidal Roig

**Affiliations:** 1 Department of Chemical Engineering, Facultad de Ciencias y Tecnologías Químicas, University of Castilla-La Mancha, Avda. Camilo José Cela 12, 13071 Ciudad Real, Spain; encarnacion.cruz@uclm.es (E.C.S.); teresa.garcia@uclm.es (M.T.G.); ignacio.gracia@uclm.es (I.G.); juan.rromero@uclm.es (J.F.R.); jesusmanuel.garcia@uclm.es (J.M.G.-V.); 2 Department of Medical Sciences, Microbiology Area, Facultad de Medicina, University of Castilla-La Mancha, Paseo de Moledores s/n, 13071 Ciudad Real, Spain; msillescasf@sescam.jccm.es; 3 Department of Microbiology, Hospital General Universitario de Ciudad Real, Obispo Rafael Torija s/n, 13005 Ciudad Real, Spain

**Keywords:** lavender essential oil, antimicrobial activity, chitosan/alginate membranes

## Abstract

The demand for natural products in the treatment of dermatological pathologies has boosted the use of bioactive substances such as lavender essential oil (LEO), which stands out for its anti-inflammatory and antioxidant properties and its antimicrobial potential. Biopolymers such as chitosan (CHT) and alginate (ALG) are biodegradable and biocompatible and have proven their viability in biomedical applications such as skin regeneration. The inhibitory effect of LEO on the growth of skin-related bacterial species *Staphylococcus aureus*, *Staphylococcus epidermidis*, *Pseudomonas aeruginosa* and the fungus *Candida albicans* was studied by incorporating 1% *v/v* LEO encapsulated in CHT, ALG, and CHT/ALG membranes. Despite the verification of the antimicrobial effect of all type of membranes, no synergistic effect was observed following the addition of LEO. *S. aureus* and *P. aeruginosa* showed the most growth on the different substrates and *C. albicans* demonstrated the highest inhibition. This is a first approach using microorganisms isolated from clinical samples or skin microbiota. Further investigation would be advisable using more clinical strains for each microorganism to validate their biomedical applicability.

## 1. Introduction

Skin anomalies are a leading epidemiological burden worldwide, ranking fourth with respect to the number of diseases they cause [1]. Skin is a complex organism inhabited by bacteria, fungi, and viruses. These microbes are essential for the proper functioning of the human body. Diseases or pathologies can, however, result from their disruption or interaction with other microorganisms or hosts [2]. *Staphylococcus epidermidis* (*S. epidermidis*) and *Staphylococcus aureus* (*S. aureus*), both gram-positive cocci, are naturally present on the skin and in the mucous membranes of the nose [3,4]. *S. epidermidis* is present in 100% of humans, while *S. aureus* is present in a lower percentage [5]. The gram-negative bacillus *Pseudomonas aeruginosa* (*P. aeruginosa*) is also present, but is less abundant and frequently behaves as an opportunistic pathogen causing burn wound infections [6] and skin and soft tissue injuries [7]. One of the most common fungI in the human microbiota is *Candida albicans* (*C. albicans*), which asymptomatically colonizes numerous parts of the body, including the gastrointestinal and genitourinary tracts of healthy people [8,9].

The demand for products of natural origin in all areas driven by new life patterns, as well as the side effects of traditional medicines for the treatment of dermatological disorders, have boosted the presence of natural extracts, such as essential oils, in the pharmaceutical industry [10]. Lavender essential oil (LEO) has been one of the most popular essential oils since ancient times. It has been traditionally used as a natural remedy in aromatherapy and herbal medicine. Currently, it is present in cosmetics, perfumes, and, particularly, in nutraceuticals and pharmaceuticals [11]. This is due to LEO therapeutic attributes, such as its anti-inflammatory and antioxidant properties and its beneficial effects on diseases related to the nervous system [12,13]. It also plays an important role in products related to the treatment of stress or anxiety or in aromatherapy to alleviate various pathologies [14,15].

Regarding the biological activity of LEO, its antifungal and antibacterial capacities are also noteworthy. This essential oil, isolated or encapsulated in various materials, has shown antibacterial activity against bacteria such as *S. aureus* and *Escherichia coli* as well as a yeast, *C. albicans* [16,17]. Consequently, LEO is a very interesting component at the pharmacological level for biomedical applications such as products for the treatment of skin diseases.

In addition to the use of high added-value compounds from natural resources, environmental issues are of great relevance to biomedicine. For this reason, biopolymers are the most commonly used materials for the encapsulation of bioactive substances [18]. Biopolymers are a type of polymers synthesised by living organisms, including natural polymers present in the environment or those produced artificially from natural resources. These materials are readily available, inexpensive, and stand out for their biodegradability and biocompatibility [19].

The most widely used in health-related applications are polysaccharides because of their biological activity, especially alginate (ALG) and chitosan (CHT). These biopolymers, apart from their ability to form membranes, have antimicrobial capacity, antioxidant potential, and even possess the ability to accelerate healing [20,21,22,23]. The combination of both polymers has been shown to work as an effective pathway for skin regeneration [24,25,26,27], with the incorporation of essential oils able to increase their biological activity and therapeutic potential [28,29,30,31,32]. In previous research, the cell viability and biocompatibility of CHT/ALG + LEO membranes with HaCaT, a human epidermal keratinocyte cell line, has been demonstrated [33]. 

In this study, ALG, CHT, and CHT/ALG membranes were prepared with the addition of LEO. Because these membranes are created with the aim of improving wound healing and curing skin pathologies, bacteria present in the human microbiota and residing on the skin and/or mucous membranes were used to test their antimicrobial capacity, namely *S. epidermidis*, *S. aureus*, *P. aeruginosa*, and *C. albicans*. While *S. aureus* and *P. aeruginosa* are mainly responsible for chronic skin infections [34], *S. epidermidis* and *C. albicans* have not been frequently associated with skin infections, but have been sporadically implicated in post-surgical infections [8,35]. Considering that biopolymers may have a bactericidal effect, the addition of LEO as an extra bactericidal additive should reflect a synergy. The aim of this work is to provide a preliminary result of the possible synergistic inhibition effect of LEO-additivated polymers using clinical strains implicated in wound or skin microbiota infections. The results obtained in this study provide a reference for further study of the antibacterial mechanism of LEO in conjunction with the proposed biopolymers and possible interactions between them.

## 2. Materials and Methods

### 2.1. Preparation of Membranes

#### 2.1.1. Materials

CHT and Tween 80^®^ were supplied by Sigma-Aldrich (St. Louis, MO, USA). Sodium alginate, ALG, from brown algae was obtained from Fluka-BioChemika (Buchs, Swizerland) and Calcium chloride from PanReac AppliChem (Barcelona, Spain). The LEO was from Peñarrubia del Alto Guadiana S. L. (Albacete, Spain). Acetone, supplied by LabChem (Santo Antão do Tojal, Portugal), acetic acid from Carlo Erba Reagents (Milan, Italy), and deionized water were also used. A total of 20 mM PBS pH 7.4 (including milli Q water, Na_2_HPO_4_·7H_2_O, and NaH_2_PO_4_·H_2_O) was used.

#### 2.1.2. Membranes Synthesis Procedure

Six types of membranes were prepared, two with a single polymer (Alginate, ALG; Chitosan, CHT) and a third mixed (CHT–ALG), combined with and without lavender essential oil (LEO).

Membranes were prepared as described in other work [33] using the method of Rodrigues et al. [25]. For the preparation of the CHT/ALG membranes, 90 mL of an aqueous solution of ALG at 0.5% (*w*/*w*) was added to 90 mL of CHT solution at 0.5% (*w*/*w*) in 2% aqueous acetic acid (*v*/*v*) and acetone 1:1 (*v*/*v*) using a syringe pump (KDS Legato 200 Series) with a flow rate of 40 mL/h and stirring at 500 rpm.

The tests were conducted in a glass vessel at a temperature of 25 °C with a mechanical stirrer. After obtaining the suspension, it was homogenized for 10 min while being stirred at 1000 rpm. The pH was then raised to 5.28 by adding NaOH (1 M), which was then agitated for 10 min at 1000 rpm. For cross-linking, 1.8 mL of a 2% (*w*/*v*) aqueous CaCl_2_ solution was added last. The mixture was then put into Petri dishes with a 15 cm internal diameter and dried for 20 h at 37 °C in an oven with moving air. Membranes were dried before being submerged for 1 h in 150 mL of a 2% (*w*/*v*) CaCl_2_ aqueous solution to cross-link alginate L-glucuronic acid residues on neighboring chains that were not attached to chitosan. They were then submerged twice for 1 h in 200 mL of deionized water, and then allowed to air dry at room temperature. 

In the case of membranes composed of a single biopolymer, the same procedure was followed, although the first step of mixing the solutions was excluded. The only difference between CHT and ALG membranes is that ALG membranes do not require pH neutralization. For the preparation of ALG, CHT, and CHT/ALG membranes with essential oil, LEO was added to the initial solution with a concentration of 1% (*v*/*v*), as well as Tween 80^®^ 1% (*v*/*v*) which acts as an emulsifying agent for the dispersion and solubilization of the essential oil [36].

#### 2.1.3. Sterilization of Membranes

Membranes were cut into square shapes with a mean area of 0.25 cm^2^. They were then sterilized by ultraviolet irradiation on both sides in a laminar flow cabinet for 1 h on each side in sterile Petri dishes. Once sterilized, they were stored in the sterile plates until use, within 72 h. 

### 2.2. Antimicrobial Capacity Analysis

This study was carried out in two stages: prior to the study of the antimicrobial capacity of the membranes synthesized with the different polymers and LEO, the inhibition capacity of the LEO itself was determined (Experiment 1). Once the behavior of the LEO against the strains used had been checked, the antimicrobial activity of the membranes was analyzed using two methods, culturing by direct contact with a solid medium (Experiment 2) and by immersion in culture broth (Experiment 3).

#### 2.2.1. Strains and Culture Media

Table 1 shows the strains used. All of them came from the area of Microbiology of the Faculty of Medicine of Ciudad Real of the UCLM as collection strains of the area for laboratory practice in the subject of Microbiology in the Degree of Medicine.

Two types of culture media were used, a solid media Tryptone Soy Agar (TSA) and a broth Tryptone Soy Broth (TSB) (Scharlab, Spain). Tryptone Soy Agar (TSA) is a versatile, non-selective medium that offers enough nutrients to support the development of a broad range of pathogenic microorganisms. It was decided to use TSA because it is a medium where the micro-organisms to be tested grow optimally.

#### 2.2.2. Inoculum Solutions

For both the measurement of the antimicrobial capacity of the oil and the membranes, the inocula for each of the strains used were prepared in the same way.

**Figure 1:**
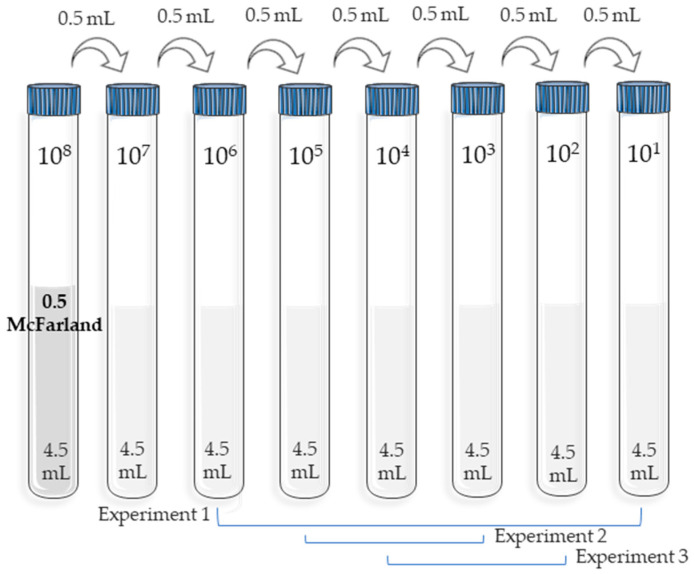
Serial dilutions of the bacterial inocula.

A schematic of the procedure followed is shown in Figure 1. A set of 10-fold serial tube dilutions was performed under aseptic conditions with 4.5 mL of sterile NaCl solution (0.9%). The initial dilution was the one with the highest microbial concentration, measured with a McFarland 0.5 standard (bioMérieux, Craponne, France) equivalent to 10^8^ CFU/mL. In parallel, for each experiment, plate counts with medium were performed to determine the starting CFU.

#### 2.2.3. Determination of Bactericidal Effect of LEO

First, TSA medium was prepared for distribution in sterile 90 mm Petri plates (Scharlab, Barcelona, Spain). Once the medium was prepared and prior to sterilisation, LEO was added. After sterilising the prepared medium with the essential oil in the autoclave, it was distributed in sterile Petri dishes used in the microbiological experiment. It should be noted that, at this stage, in order to obtain a homogeneous distribution of the LEO in the medium, continuous magnetic stirring of the container was performed.

In order to test the influence of LEO concentration on its antibacterial activity, different concentrations of 1, 0.75, 0.5, 0.25, 0.1, and 0% *v*/*v* were prepared, the latter serving as control. In addition, different concentrations of inoculum of each strain were also used (from 10^8^ to 10^1^). A total of 10 µL of each of the dilutions was inoculated in quadruplicates on a TSA plate, from dilution 10^6^ to 10^1^, and left to incubate at 35–37 °C for 18–24 h. After this time, colony counts were conducted for the *S. epidermidis*, *S. aureus*, and *P. aeruginosa* tests. In the case of *C. albicans*, the count was done at 48 h of incubation.

#### 2.2.4. Microbiological Study of Direct-Contact Membranes in Solid Media

**Figure 2:**
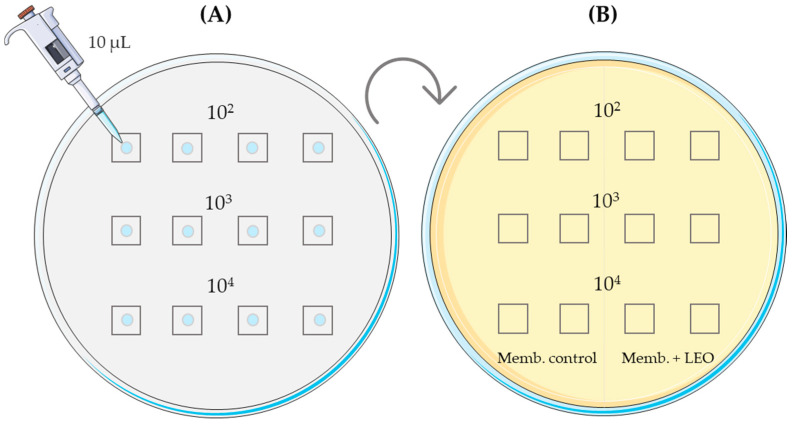
(**A**): placement of the inoculum on the membranes in a sterile Petri dish; (**B**): culture of the inoculated membranes in the TSA medium.

In this case, the 10 µL inoculum was cultured directly onto the membrane pieces and placed in contact with the TSA medium on the plates, previously prepared as in the previous experiment (Figure 2A). Three different inoculum concentrations (10^3^, 10^4^, and 10^5^) were used, combined with three different times (from inoculum application on the membranes to incubation on the culture plate) at 0 h, 2 h, and 6 h. Each test was duplicated on the same plate, comparing the control with the membrane, including LEO (Figure 2B), for each type of membrane synthesized, CHT, ALG, and CHT/ALG. Plates were incubated in aerobic conditions at 35–37 °C for 18–24 h for all three bacteria and at 48 h for *Candida*. After incubation, plates were analyzed for the presence or absence of microbial growth.

#### 2.2.5. Microbiological Study of Membranes in Broth

The key difference between this method and the contact experiment is that, after the membrane inoculation phase, instead of placing the membranes on TSA solid medium plates, they were immersed in TSB liquid medium for 24 h in sterile 24-well plates (Deltalab, Barcelona, Spain). Inoculation of the membranes was attained following the same method used in the contact experiment, and, in this case, the used concentrations were 10^2^, 10^3^, 10^4^ CFU/mL. As in the experiment in the solid medium, membranes were grown on the plates at three different times after the inoculum was placed on them: at 0 h, 2 h, and 6 h.

**Figure 3:**
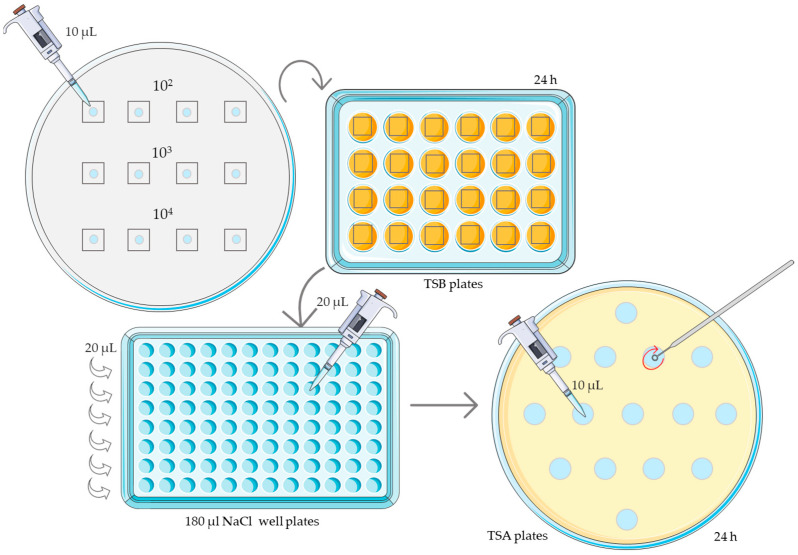
Procedure for the culturing of membranes in broth and subsequent colony counting.

After 24 h, 20 µL of liquid medium was taken from each well. In this experiment, not only the presence or absence of microbial growth was analyzed, but also a CFU/mL count was performed using 10-fold serial dilutions and 0.9% NaCl as dilutant. These dilutions were carried out in sterile 96-well plates (Deltalab, Spain) in aseptic conditions. A total of 10 µL of each dilution were taken and cultured on TSA plates and left to incubate at 35–37 °C for 18–24 h (Figure 3). After this time, colony counts were performed for the *S. epidermidis*, *S. aureus*, and *P. aeruginosa* tests. In the case of *C. albicans*, the count was done at 48 h.

#### 2.2.6. Database and Statistical Analysis

A database was created using Microsoft Excel 2021, with the following variables included: membrane type (ALG, CHT, CHT/ALG, ALG + LEO, CHT + LEO, CHT/ALG + LEO), experiment (1,2,3), strain (*C. albicans*, *P. aeruginosa*, *S. aureus*, *S. epidermidis*), inoculum (10^6^, 10^5^, 10^4^, 10^3^, 10^2^, 10^1^), time (0 h, 2 h, 6 h), growing (NEG, POS), count (only for Experiment 3: 0, <100, 100–1000, >1000). To evaluate significant differences between qualitative discrete variables, the Pearson’s Chi-square (χ^2^) test was used, with a statistical significance level of *p* < 0.05, using the Winepi program (www.winepi.net, (accessed on 2 October 2023); basic statistics, Ignacio de Blas, University of Zaragoza). As there was a low sample size for each experiment, different variables such as total strains, pre-inoculum times, or membrane type (with or without LEO) were grouped together, since, in most of the analyses, the expected results in the test were lower than 5, rendering invalid the significance of χ^2^ test.

## 3. Results 

### 3.1. Bactericidal Effect of LEO

The objective of this experiment was to evaluate what concentration of LEO is sufficient to inhibit microbial growth or produce changes in the phenotype of the colonies, which could be indicative of a microbial stress effect, by cultivating the different strains in a solid medium supplemented with the essential oil.

Table 2 shows the average count results adjusted for inoculum dilution 10^3^ for the strains studied. The main finding was that the 1% *v*/*v* LEO concentration inhibited the growth of all strains. The count was similar between control and LEO dilutions lower than or equal to 0.5%. The 0.75 *v*/*v* LEO concentration also inhibited *S. epidermidis* and *C. albicans*, while for *S. aureus* and *P. aeruginosa*, although there was growth, this concentration subtly reduced the number of colonies.

**Figure 4:**
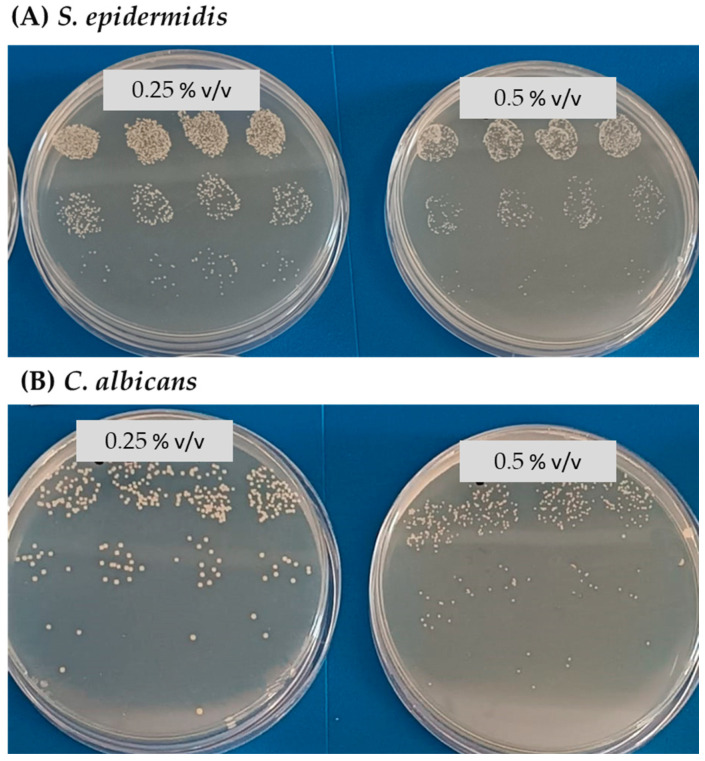
Colony phenotype comparing 0.25% *v*/*v* LEO and 0.5% *v*/*v* LEO for *S. epidermidis* and *C. albicans*.

Although there were no differences in counts between 0.5% *v*/*v* LEO and the controls, a change in the phenotype was observed in the case of *S. epidermidis* and *C. albicans* (Figure 4), showing smaller colonies. This change in phenotype was not observed in either *S. aureus* or *P. aeruginosa*.

### 3.2. Growth Inhibition Depending on the Time between Inoculum Addition and Culture

**Figure 5:**
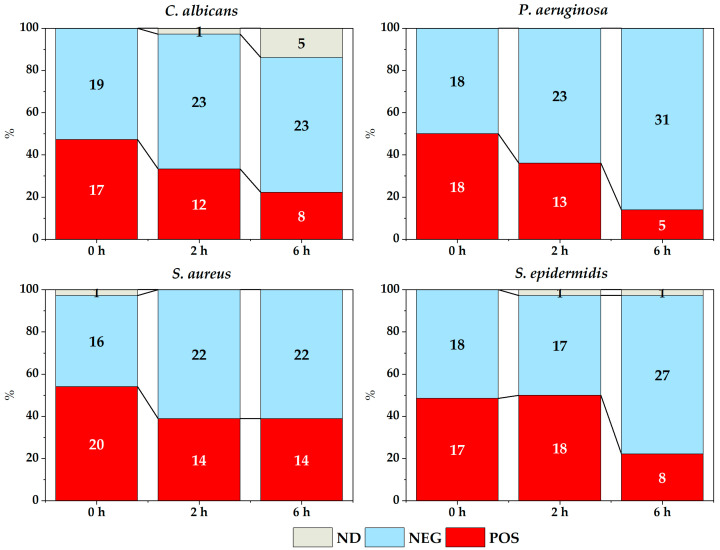
Microbial growth depending on the time elapsed between placing the inoculum in experiment of membranes in solid medium (POS: positive, NEG: negative, ND: not determined).

**Figure 6:**
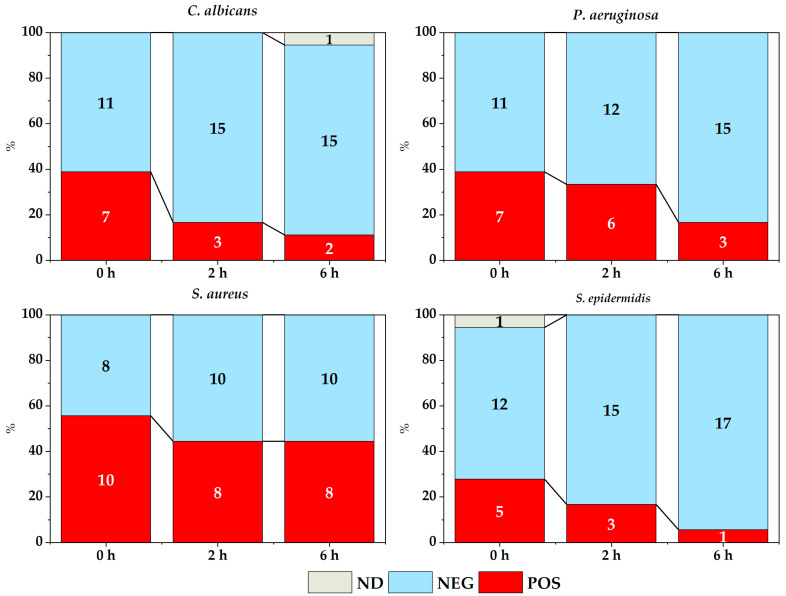
Microbial growth depending on the time elapsed between placing the inoculum in the experiment of membranes in broth (POS: positive, NEG: negative, ND: not determined).

Figure 5 and Figure 6 show the count of membranes with and without microbial growth depending on the time elapsed between placing the inoculum on the different membranes and its corresponding culture, in both the experiment in solid medium (Figure 5) and the experiment with broth (Figure 6).

Overall, there was an effect of the time elapsed between inoculum addition and subsequent cultivation for almost all strains studied, both in the contact experiment (Figure 5) and in the broth experiment (Figure 6), except for *S. aureus* in both cases. For this strain, it should be noted that the number of positive cases remained constant for the experiment in the solid medium (χ^2^ = 2.700, *p* = 0.2592) and also in the experiment with membranes in broth (χ^2^ = 1.637, *p* = 0.4412), except for a slight decrease of positive cases in the broth over time.

### 3.3. Bacterial Growth Resulting from Membranes Cultured Directly on Plates 

**Figure 7:**
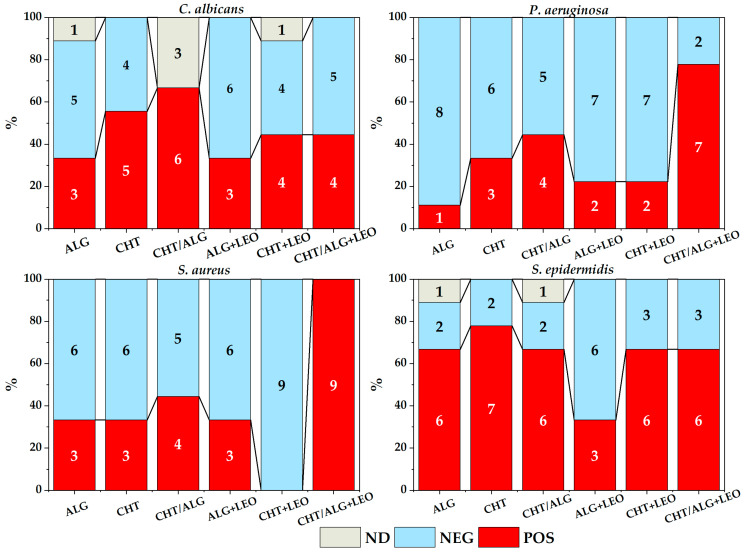
Summary of bacterial growth for each strain and membrane type for experiment of direct contact in solid medium (POS: positive, NEG: negative, ND: not determined).

Figure 7 summarizes all the results of experiment of direct contact, for each type of membrane and strain. In some cases, it was not possible to obtain a result because the membranes were detached from the culture medium or there was some type of contamination, mainly in the oil-free membranes.

To evaluate the effect of membrane oil addition (LEO), all strains and membranes with and without LEO were grouped, but no significant differences were observed (χ^2^ = 0.370, *p* = 0.5429). On the other hand, upon separation by type of membrane, no differences were found either, although there was a slight tendency of greater inhibition in CHT membranes with LEO (χ^2^ = 1.796, *p* = 0.1802). 

In addition, the difference in total membranes was evaluated for each of the strains (χ^2^ = 11.6, *p* = 0.0089), concluding that *S. epidermidis* was the best-growing strain.

### 3.4. Bacterial Growth Resulting from Membranes Cultured in Broth 

**Figure 8:**
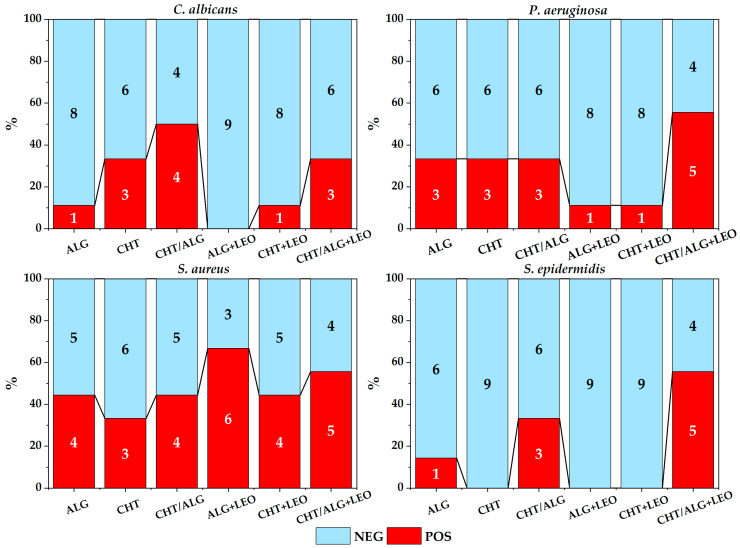
Summary of bacterial growth for each strain and membrane type for the experiment involving membranes in broth (POS: positive, NEG: negative).

Figure 8 summarizes all the results of experiment in broth, for each type of membrane and strain.

In this experiment, in addition to assessing the presence or absence of growth, counts were made from the culture broth (CFU/mL). Although it was not possible to analyze significant differences in counts for each strain individually due to the small sample size (less than 5 samples), for *C. albicans* further growth was observed on CHT/ALG membranes; also, by comparing membranes with or without LEO, the number of positive cases was found to be slightly higher when LEO was included.

No significant differences were obtained by grouping the results of counts with respect to the presence of LEO in the total strains (χ^2^ = 0.080, *p* = 0.7769), but there were differences in growth among strains (χ^2^ = 13.951, *p* = 0.0029), with *S. aureus* and *P. aeruginosa* growing better in broth than *C. albicans* and *S. epidermidis* (χ^2^ = 9.8945, *p* = 0.0016).

Furthermore, by grouping all strains together, no differences in positivity were found in the total number of membranes (χ^2^ = 0.080, *p* = 0.7769) or with respect to each membrane type. By comparing strains, *S. aureus* was found to be the strain with the highest number of positive cases, with no differences when LEO was used.

By grouping by the type of polymer used between oil and non-oil membranes, it was observed that there was a slightly higher growth in mixed membranes with respect to all strains (χ^2^ = 11.0652, *p* = 0.0039), with the exception of *S aureus*.

**Figure 9:**
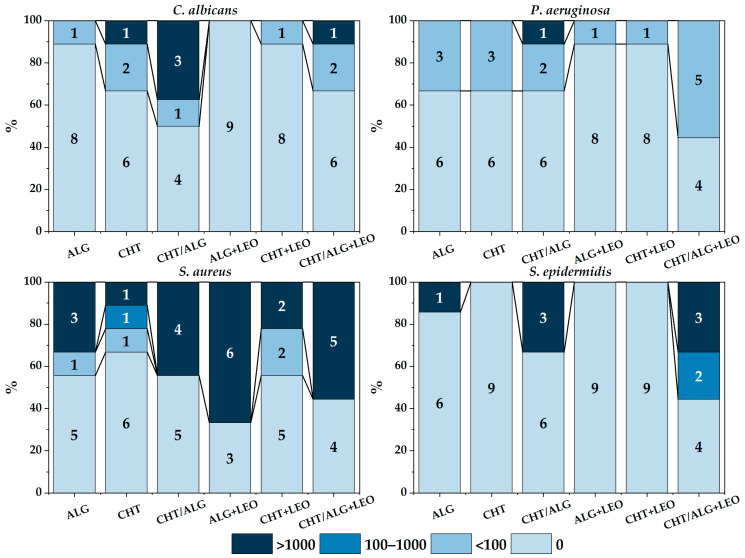
Summary of the number of colonies determined for each membrane and strain in experiment in broth.

Figure 9 breaks down the number of positive colonies into intervals in order to really see the influence of LEO on the total percentage of inhibition of each of the strains. It can be seen that *S. aureus* shows less difference in the number of positive colonies between the membranes with or without LEO and between the biopolymers used.

## 4. Discussion 

The study of the concentration of LEO that does or does not inhibit the growth of the microbial strains chosen for this study, *C. albicans*, *P. aeruginosa*, *S. aureus*, and *S. epidermidis*, provides preliminary information on how the addition of this essential oil can influence different biopolymers for biomedical applications. In this work, the antimicrobial effect has been analyzed using strains isolated from clinical cases or from the microbiota itself, trying to demonstrate real cases, as other researchers also do using LEO, with environmental strains from hospital settings [37] or with different strains of a single species from clinical isolates [38].

Initially, it was found that LEO inhibited the growth of all micro-organisms tested at 1% concentration in the medium. However, in the case of *S. epidermidis* and *C. albicans* total inhibition already occurred at a concentration of 0.75%. Although no changes in colony counts were observed at 0.5% *v*/*v* LEO for *S. epidermidis* and *C. albicans*, changes were observed in the phenotype of the colonies, which grew smaller and with a rougher appearance. This phenotypic change may indicate possible cellular alterations. In general, the antimicrobial power of essential oils is related to changes in membrane permeability, as oils penetrate membrane lipids, destroying them and making them more permeable [39]. With respect to *S. aureus*, in a study using aguaribay oil, in addition to increasing membrane permeability, an ability to inhibit biofilm formation was demonstrated [40]. Again with respect to *S. aureus*, other research using clove essential oil demonstrated, by electron microscopy, morphological changes at the cellular level [41]. In particular for *S. epidermidis*, the use of Zanthoxylum schinifolium oil not only resulted in changes in membrane permeability and integrity, but also in alterations in the physiological functions of the bacterium [42].

According to the literature, it is evident that essential oils have antibacterial effects, and, among all of them, LEO has been extensively studied [43]. In this case, it has been observed that the higher the concentration of LEO, the higher the degree of inhibition of the four strains studied, as it has also been observed in other works [44]. However, in experiments testing membranes, we did not observe differences between the addition or non-addition of LEO, unlike other researchers who have observed more antimicrobial efficacy when LEO was added at concentrations of 0.5% *v*/*v* and 1% *v*/*v* using a carboxymethylchitosan-based substrate [41]. On the one hand, the biopolymers themselves already have an antimicrobial effect [41,45,46]. In fact, this work shows that the mixed CHT/ALG membrane has proven to be the least inhibitory, perhaps due to the dilution effect of both polymers in the membrane which has already been observed to have a lower toxicity effect in cell cultures compared to single-component polymers [33]. On the other hand, because LEO in the biopolymer is less bioaccessible, it is necessary to analyze the surface of the membranes to revise the composition or to add LEO in the form of nanoemulsions as in other works [47,48]. Another possible explanation could be a low concentration of the oil used, as other studies have found more inhibition at higher concentrations of the essential oil, as in the case of LEO [44] or other essential oils [49].

In general, differences in inhibition were found depending on the experiment performed. It is difficult to make comparative studies with what is available in the scientific literature. Most published works use disk diffusion techniques [27,38,50] or MIC techniques [44,46,50,51]. In this case, two experiments have been performed that aim to get closer to reality by using a dressing on a wound. On the one hand, transferring the wound to the culture medium itself (solid or broth) with the dressing on it, on the other, analyzing different inoculum concentrations, as these microorganisms reside in the normal skin in low concentrations prior to a skin infection.

Another consideration is that it is difficult to determine differences between microbial species, as there may be differences caused by usage of different strains of the same microbial species [38] and even between two collection strains of the same species with different antibioresistance profiles [37]. Overall, in the present work if there is growth inhibition in all the strains studied, with or without LEO.

*S. aureus* and *P. aeruginosa* have grown the most on the different substrates. Although these two bacteria are not very frequent in the skin microbiota, they have great clinical repercussions and are the ones that colonize most skin wounds, thus causing chronic infections [34]. However, they did show growth inhibition in all the membranes tested. In the experiment involving contact between the biopolymer and the solid culture medium, *S. epidermidis* was found to be the strain that obtained the most positive cases, but this was not the case when it was cultured in broth. The broth culture experiment was more inhibitory than the contact experiment. This may be due to a release of both the oil and the biopolymer itself into the liquid culture medium, as the membranes were partially degraded after incubation. In the case of *C. albicans*, in the present work the fungus suffered overall more inhibition than the bacteria. In contrast to other work using LEO, there was almost no inhibitory effect using also a strain isolated from a clinical case [50], nor using a reference strain, with a null [44].

## 5. Conclusions

This work provides information on the preliminary study of the antimicrobial potential of LEO and its incorporation into CHT and ALG membranes. Firstly, it can be deduced that LEO has an inhibitory capacity in a concentration of 1% *v*/*v* in TSA medium with respect to *P. aeruginosa* and *S. aureus*, as well as with respect to *S. epidermidis* and the fungus *C. albicans* from a concentration of 0.75% *v*/*v*, with *C. albicans* suffering an alteration in the phenotype of the colonies. Despite the verification of the antimicrobial effect of LEO, in the membrane tests, in general, no significant differences were observed between the results obtained with and without its addition. Furthermore, the CHT/ALG membrane proved to be the least inhibitory, probably due to the dilution effect of both polymers in that configuration. As for the difference between microbial species, differences were reflected with *S. aureus* and *P. aeruginosa* showing the most growth on the different substrates despite demonstrating some inhibition on all membranes. It was the fungus *C. albicans* that demonstrated the highest inhibition. The main limitation of this study is the few strains analyzed and the low number of replicates. Expansion of the experiments through an increase in the LEO concentration in membranes, along with the introduction of different variants of each specie could yield broader outcomes.

## Figures and Tables

**Table 1:** Microbiological strains used.

Bacterial Species	Source
*Staphylococcus aureus*	Strain isolated in 2017 from the nasal cavity of a human carrier, as part of its saprophytic microbiota.
*Pseudomonas aeruginosa*	Strain isolated in 2022 from a clinical case from the microbiology department of the Hospital General Universitario de Ciudad Real.
*Candida albicans*	Strain isolated in 2017 from a clinical case from the microbiology department of the Hospital General Universitario de Ciudad Real.
*Staphylococcus epidermididis*	Strain isolated in 2017 from skin microbiota of a human carrier, as part of its saprophytic microbiota.

**Table 2:** LEO inhibition in different strains expressed as mean number of colonies at a concentration of 10^3^ CFU/mL.

Strain	Control	0.1% *v*/*v* LEO	0.25% *v*/*v* LEO	0.5% *v*/*v* LEO	0.75% *v*/*v* LEO	1% *v*/*v* LEO
*C. albicans*	1184	2090	1613	1488	0	0
*P. aeruginosa*	1850	2325	2300	3230	950	0
*S. aureus*	8150	7100	14000	11650	4350	0
*S. epidermidis*	1800	1775	1033	950	0	0

## Data Availability

The data presented in this study are available upon request from the corresponding author. The data are not publicly available due to privacy.
